# Pants on fire: Risks for and outcomes of atypical lying

**DOI:** 10.1002/jad.12441

**Published:** 2024-10-29

**Authors:** Romain Decrop, Meagan Docherty

**Affiliations:** ^1^ Department of Psychology Bowling Green State University Bowling Green Ohio USA

**Keywords:** lying, offending, prolific lying, psychopathy, substance use

## Abstract

**Introduction:**

Most people are generally honest, but around 5% of individuals are prolific liars, some of whom lie for fun or no reason. However, developmental research on atypical lying features (e.g., motives, attitudes, inclinations for dishonesty) and the associated traits and negative outcomes is lacking. We examined how psychopathic traits are related to the development of lying trajectories and whether exhibiting atypical lying features during a developmental period when lies tend to decrease in frequency (i.e., adolescence to adulthood) increases the risk for adulthood antisocial behaviors.

**Methods:**

Data come from the multisite Pathways to Desistance project, a longitudinal study of serious juvenile offenders in the United States who were interviewed across 11 time points over 7 years from 2000 to 2010. Age‐based trajectory analyses modeled self‐reported atypical lying features from ages 14 to 26 for male participants (*N* = 1170; 42.1% Black, 34.0% Hispanic, 19.2% White, 4.6% Other), and examined how subscales from the Youth Psychopathy Inventory predicted lying trajectory classes and whether those classes differed in adulthood offending and substance use.

**Results:**

Around 5% of the sample maintained elevated atypical lying features in adolescence and into adulthood. These individuals were more manipulative, remorseless, impulsive, and irresponsible in adolescence, and were more likely to offend and use substances in adulthood.

**Conclusions:**

Findings highlight how atypical lying features during the normative developmental desistance period of lying may be elevated for prolific liars and how traits can be used to identify at‐risk individuals. This information will help to inform intervention and prevention programs targeting externalizing behaviors.

## 1 INTRODUCTION

Lying has been used throughout history for both personal gain and altruistic purposes (e.g., Ponzi schemes, hiding Jews during the Holocaust; Talwar & Crossman, 2011). Despite society decrying lying as inappropriate, often leading to negative ramifications (e.g., tarnishing character and relationships, causing legal and financial problems; Curtis & Hart, 2022b), situations exist where it is socially encouraged to remain polite and protect others' feelings. Some even argue for lying's inescapable necessity (Hutchins, 2022; Nyberg, 1993), particularly to foster and maintain relationships (Broomfield et al., 2002; DePaulo et al., 1996). Indeed, studies have found that lying is a normative and ubiquitous developmental behavior observed in similarly aged children across cultures (Lee & Imuta, 2021; Sai et al., 2021). However, there are cultural differences in motives for lying in adults (Choi et al., 2011; Marett et al., 2017), with little research examining racial differences in lying motives. Regardless, given that this paradoxical behavior can be both desirable and reprehensible, selfish and selfless, adaptive and maladaptive, and harmful and uplifting, some struggle to master its social propriety.

Given this complexity, studies have unsurprisingly found positive associations between children's lying and their cognitive development. Specifically, children must develop theory of mind (i.e., ability to attribute mental states to others; ToM), executive functions (e.g., inhibitory control) and social skills (e.g., communication, interpretation of norms) to successfully lie (Byrne & Whiten, 1992; de Waal, 1992; Decrop & Docherty, 2024; Talwar, 2018). However, there are mixed findings regarding the relationship of lying with intelligence (Curtis & Hart, 2022b; Stouthamer‐Loeber, 1986), with some finding an association with emotional intelligence (Porter et al., 2011) but not general intelligence (Michels et al., 2020). Nevertheless, Talwar and Crossman (2011) investigated the emergence of lying in early childhood and discovered that children's understanding of lies start as statements that are simply incorrect, but grows in complexity to include intention and the lie's social impact and acceptability.

Around two to 3 years old, children start making self‐serving antisocial lies (e.g., to avoid punishment, gain benefits) once they develop a sense of self and a rudimentary ToM. In doing so, they seek to protect themselves and begin to understand that others are not aware of their own private thoughts. However, it is not until around 7 years old that children acquire the cognitive abilities to maintain a lie's plausibility by ensuring that subsequent statements do not contradict it (Talwar & Crossman, 2011). Then, as children learn social etiquette, they move away from antisocial lies and begin using prosocial ones to maintain relationships and conform to norms (Decrop & Docherty, 2024; Hutchins, 2022; Serota et al., 2022). However, not everyone follows this pattern, and some continue to lie for fun or no reason (Levine et al., 2016). These are highly atypical motives given that normative lying is rooted in opportunity‐based‐incentives, such that people have an “honesty default” that is overridden when rewards outweigh the risk (Levine, 2020).

In terms of lying frequency, normative lie‐telling increases throughout childhood and early adolescence, before decreasing from adolescence into adulthood from socialization processes (e.g., parenting), as adolescents learn to interact with their environment without the need to frequently lie (Debey et al., 2015; Levine et al., 2013; Talwar, 2018). However, human observation is no better than chance at detecting lying (Bond & DePaulo, 2006). Thus, good liars may lack the deterrent factors that typically facilitate the socialization process when caught, leading to rewards of personal gain (e.g., excitement, material benefits) sans punishment. This may encourage the behavior through conditioning and induce lying as a “go‐to” strategy.

This maladaptive development of lying has been observed in both adults and at‐risk youth, where a small number of prolific liars ( ~ 5%) end up telling most of the lies (Curtis & Hart, 2022b) starting as early as 7 years old (Gervais et al., 2000). Specifically, individuals who tell more than three to five lies per day can be labelled as prolific liars (Curtis & Hart, 2022b; Serota & Levine, 2015), though there are subgroup differences. For example, Curtis and Hart (2022b) argue that pathological liars are a subset of prolific liars whose lies cause them significant distress and impairment in functioning. Given that around 60% of the variance in lying is explained by stable individual differences and that only around 1% of the population almost never lies (Serota et al., 2022), there is a longitudinal need to go beyond lying frequency to best understand the etiologies of prolific liars. For example, studies have shown that person‐centered latent class analyses are better for identifying at‐risk individuals than variable‐centered approaches when examining dishonesty, secrecy, disclosure, and lying (e.g., Baudat et al., 2022; Elsharnouby & Dost‐Gözkan, 2020). However, without the longitudinal component to capture chronicity, these studies can neither distinguish between prolific liars and a normative “bad lying day” (Serota et al., 2022) nor highlight potential features influencing the etiology of deceptive tendencies.

Nevertheless, frequent and persistent lying has long been associated with antisociality, and this association becomes stronger with age (Achenbach & Edelbrock, 1979; Gervais et al., 2000; Stouthamer‐Loeber, 1986). Specifically, lying is one of the diagnostic criteria for antisocial personality disorder in the DSM‐5 (American Psychiatric Association, 2013), is a hallmark feature of psychopathy (Hare & Neumann, 2005), and is frequently used after substance use to hide use and avoid punishment or shame (Farber et al., 2019; Ford, 1996). Interestingly, studies have found that although youth with externalizing behavior problems are more likely to lie for personal gain (Liu et al., 2022), they are less skilled at it (Talwar & Lavoie, 2022). Put together, it appears that the development of lying, offending, and substance use are partly a function of cognitive and social immaturity that emerge from underdeveloped behavioral strategies (e.g., self‐regulation, problem‐solving, coping skills; Talwar & Crossman, 2011) and individual traits (e.g., impulsivity; Dykstra et al., 2023; Murray et al., 2018) that may interfere with the normative desistance of the behaviors into adulthood. Nevertheless, the temporal ordering of offending and substance use with lying is unclear, and although both antisocial behaviors lead to more lying to cover them up, less is known regarding these outcomes as ramifications of atypical lying features (e.g., motives, inclinations for dishonesty). Therefore, studying lying in antisocial youth during developmental desistance periods may illuminate the traits associated with the development of prolific lying and resulting risks.

Despite lying's longstanding literature, research has overlooked key features of prolific liars. Specifically, given that lying is generally measured as a frequency using experimental paradigms, cross‐sectional samples, or retrospective reporting (Gerlach et al., 2019), no studies have investigated changes in atypical motivations for lying (e.g., amusement, no reason) across lying's normative desistance stage (i.e., adolescence to adulthood). This is needed since more favorable attitudes towards lying have been found to be one of the strongest predictors of lying (Buta et al., 2021; Hart, Jones, et al., 2019), since the factors affecting the maintenance of lying differ from those influencing its onset (Stouthamer‐Loeber, 1986), and since this developmental period may bridge the maintenance of prolific lying into a persistent life‐course behavior.

Additionally, there is currently no reliable way to predict which children will become prolific liars as adults (Talwar & Crossman, 2011) since most studies focus on external factors (e.g., parenting; Ma et al., 2015; Talwar et al., 2017) and cognitive skills (Talwar, 2018) rather than stable individual characteristics. Thus, examining how antisocial personality traits, which remain relatively stable across the lifespan (Ferguson, 2010), predict atypical motivations for lying may help identify at‐risk youth. Specifically, since impulsivity has been linked to lying and a wide range of antisocial behaviors (Dykstra et al., 2023; Murray et al., 2018), it may be part of an underlying mechanism leading to prolific lying. Lastly, no studies have examined how developmental trajectories of atypical lying motivations at key points of the age‐crime curve (i.e., adolescence to adulthood; Moffitt, 1993) predict adulthood offending or substance use.

### 1.1 Current study

This study is the first to use a longitudinal person‐centered statistical approach (i.e., Latent Class Growth Analysis; LCGA) to examine the developmental course of atypical lying features (i.e., motives, inclinations for dishonesty) in a high‐risk sample of male offenders followed from adolescence to adulthood. We hypothesized that although most participants will follow the normative trend of lying with declines in scores into adulthood, a small percentage will maintain elevated levels. Next, because lying is a hallmark of psychopathy and psychopathic traits are relatively stable across the lifespan (Neumann et al., 2011), we examined how psychopathic traits are related to normative desistance to identify individual characteristics associated with persistent atypical lying features.

Although we hypothesized that higher maladaptive interpersonal (e.g., dishonest charm), affective (e.g., callousness) and lifestyle traits (e.g., impulsivity) will predict atypical lying features, given the lack of research examining these traits concurrently, we could not anticipate which specific traits would be significantly related to atypical lying features outside of impulsivity. Furthermore, we hypothesized that elevated lying scores across this developmental stage will be related to increased risk for offending and substance use in adulthood. Because lying, offending, and substance use have lower prevalence and variability in community samples, we selected a high‐risk sample to test our hypotheses. Lastly, given the inconclusive literature relating IQ and race to atypical lying features, and because both have been associated with justice system involvement (Koenen et al., 2006; Nyborg & Jensen, 2007; Rocque, 2011), we tested them as predictors of lying and antisocial outcomes. Our findings further the understanding of the development of lying by identifying characteristics that may impede typical desistance and point towards risks that may be common among prolific liars.

## 2 MATERIALS AND METHODS

### 2.1 Participants

Data originate from the public multisite Pathways to Desistance project, a longitudinal study of United States juvenile offenders in Philadelphia, PA (*n* = 605 males) or Phoenix, AZ (*n* = 565 males; Schubert et al., 2004). Consistent with previous trajectory studies (e.g., McCuish & Lussier, 2021), only male participants (*n* = 1170) are examined in analyses given the insufficient female sample size (*n* = 184) for proper trajectory stability (Nagin, 2005) and concerns about the extension of psychopathy instruments to females (Forouzan & Cooke, 2005). Participants 14–18 years old were eligible if they were charged with a felony or serious nonfelony offense (e.g., sexual assault), though recruitment of drug‐related offenses was capped at 15% to ensure diversity in offenses.

### 2.2 Procedures

After participants provided informed consent or assent with guardian consent, two 2‐h interviews were administered at baseline, either in a confinement facility or agreed upon location. Precautions were taken to ensure comprehension and confidentiality, with the full study procedure summary in Schubert et al. (2004). Follow‐up interviews (Waves 1–10) were conducted at 6‐month intervals for a 3‐year period and then annually over the next 4 years, with high retention rates across the 11 total time points that ran from November 2000 to March 2010 (range = 84%–94%, *M* = 90%). The project is set up as an accelerated, longitudinal cohort design with a mean baseline age of 16.05 (SD = 1.16), allowing for age‐based trajectory analyses from 14 to 26. Only age rounded to the nearest integer was publicly available, and because only one participant was 19 years old at baseline, this participant was excluded from analyses to ensure adequate representation across age groups and to limit model bias, uncertainty, and error. In our analytic sample 41.1% of participants were Black, 34.0% Hispanic, 19.2% White, and 4.6% other (e.g., biracial, Native American).

### 2.3 Measures

#### 2.3.1 Youth psychopathy inventory (YPI)

The YPI (Andershed et al., 2002) was administered at waves 1 through 10, but not baseline, and consists of 50, 4‐point Likert items (ranging from 1, 'Does not apply well at all,' to 4 'Applies very well') that are summed into 10 subscales of five questions each, including: lying, dishonest charm, grandiosity, manipulation, impulsiveness, irresponsibility, thrill‐seeking, unemotionality, remorselessness, and callousness. The five YPI lying items are: “Sometimes I find myself lying without any particular reason.”, “Sometimes I lie for no reason, other than because it's fun.”, “I've gotten into trouble because I've lied too much.”, “I like to spice up and exaggerate when I tell about something.”, and “It's fun to make up stories and try to get people to believe them.” Importantly, these items do not directly assess engagement in lying (i.e., frequency), but rather atypical endorsements of a higher tendency to lie without a clear motive or for one's enjoyment or amusement, as well as an inclination for, or history of, dishonesty. The lying subscale was internally consistent (Cronbach's alphas range across waves = 0.79–0.83) and relatively stable over time (bivariate correlations from consecutive waves range = 0.47–0.59). Since the YPI was only administered to some participants at Wave 1, we created scores by averaging the nine subscales' means—excluding lying—across Waves 1 and 2, such that participants with data at both waves had a mean score from both, while those with data at only one of the two waves used that single wave's score. Subscale means, standard deviations, and reliability coefficients are in Table A1, and inter‐subscale correlations are in Table A2.

#### 2.3.2 Self‐reported offending (SRO)

The SRO (Huizing et al., 1991) consists of 24 items asking participants whether they ever participated in antisocial and/or illegal activities (e.g., damaged property, stole, sold drugs) at baseline, and whether they participated in these activities during the last recall period (i.e., 12 months) for Wave 10. Since two items were not consistently asked, a proportion variety score was created based on the remaining 22 items with the proportion of endorsed items divided by the number of questions answered. This approach is used because psychopathy is linked to versatile criminality (Hare & Neumann, 2005) and offending variety scores are more internally consistent and stable than offending frequency scores (Bendixen et al., 2003). The mean score at baseline was 0.34 (SD = 0.21, *N* = 1166) and dropped to 0.05 (SD = 0.09, *N* = 953) by the final wave.

#### 2.3.3 Self‐reported substance use

Lifetime substance use was measured via variety score such that frequencies of use for nine substances (cannabis, opiates, cocaine, stimulants, ecstasy, sedatives, hallucinogens, inhalants, amyl nitrate) were dichotomized (0 = *No use*, 1 = *Any use*) and summed (range = 0–9). The mean baseline score was 2.06 (SD = 1.91, *N* = 1164) and dropped to 0.61 (SD = 1.10, *N* = 951) at the final wave when youth were asked about their use during the last recall period (i.e., 12 months).

#### 2.3.4 Race/ethnicity

Three dichotomous race/ethnicity variables (0 = *No*, 1 = *Yes*) are included for Black, Hispanic, and other participants, making White individuals the reference group.

#### 2.3.5 Intelligence quotient (IQ)

The Wechsler Abbreviated Scale of Intelligence (WASI; Wechsler, 1999) was administered at baseline, and consists of the Vocabulary (42 orally defined items) and Matrix Reasoning subtests (35 incomplete grid patterns). These scores were combined to produce an age‐based standardized general intellectual functioning score, with higher values indicating greater ability (*M* = 84.47, SD = 12.84, *N* = 1158).

### 2.4 Analytical plan

Data are publicly available (www.icpsr.umich.edu/web/NAHDAP/studies/29961). All analyses were conducted in *Mplus* (Version 8.8; Muthén & Muthén, 2017) and figures were created in *R* (R Core Team, 2021). This study was not pre‐registered and did not need Institutional Review Board approval because secondary analysis of publicly available data does not meet the definition of human subjects research.

We first identified the lying trajectory of the full analytic sample with a latent growth curve model (Curran & Hussong, 2003) with the data aligned by age rather than wave. The age variables were centered at 20, the most populated age across waves, and predictors were centered at their grand means for interpretability of results when comparing parameters of outcomes between classes. We used a robust maximum likelihood (MLR) estimator and the “tscore” option in *Mplus* to create definition variables with age as the individually varying time of observation by wave (Mehta & Neale, 2005). We determined the best‐fitting lying trajectory by comparing models that differed in how they characterized change over time (i.e., intercept‐only, linear, and quadratic fixed and random effects). The best‐fit model was chosen based on lower relative AIC, BIC, and SABIC indices and chi‐square difference tests of nested models.

Using the best‐fit growth model, an LCGA clustered together groups of individuals with similar lying trajectories to identify individuals who were prone to more elevated levels of atypical lying features over time. Following Asparouhov and Muthén's (2014) methodology, a three‐step bias‐adjusted latent class modeling approach using a random mixture model was used to allow for the “tscores” option. After comparing up to six latent classes of growth in step one, the best‐fitting model was determined based on lower relative AIC, BIC, SABIC indices, an entropy score above 0.80, and class sizes above 5% of the sample (Nagin, 2005). Next, step two is used to determine the measurement error for the most likely class variable *N* so that it can be fixed and pre‐specified in the third step. In this final step, using Monte Carlo integration, the lying trajectory classes were regressed with a multinomial logistic regression on the nine remaining YPI subscale averages, race/ethnicity, and IQ. In the same model, Wave 10 substance use and offending were regressed on the lying class trajectories. For the former part of the model, the most populated, normative class was used as reference, and in the latter part, race/ethnicity, IQ, and either lifetime substance use or offending at baseline, based on the respective outcome, were used as control variables. Covariances were modeled among all predictor and control variables. A negative binomial regression was used for substance use because it is an overdispersed count variable (King, 1989) and thus most absolute fit indices (e.g., CFI, TLI, RMSEA) could not be calculated in *Mplus*. Instead, the adequacy of fit was determined by comparing available fit statistics (i.e., Loglikelihood, AIC, BIC, SABIC) to a null version of the same model where the predictors', covariates', and outcomes' correlations were fixed to zero. Lastly, parameters were created and compared for the offending and substance use outcomes, while holding controls at zero, for class comparisons.

## 3 RESULTS

### 3.1 Lying trajectories

**Figure A1:**
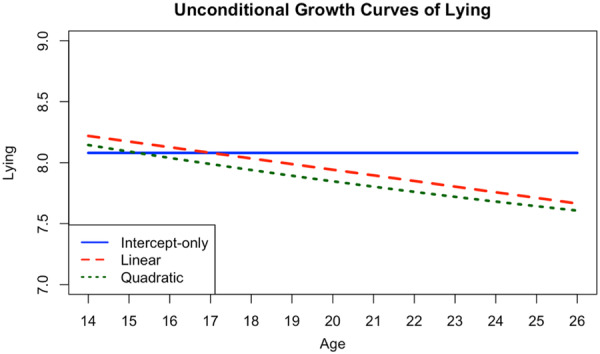
Unconditional growth curves of lying. *N* = 1152; Figure created using RStudio (R Core Team, 2021).

Results of the overall latent growth curve model comparisons are depicted in Figure A1 and revealed that the model allowing for quadratic growth best fit the data. On average, individuals at age 20 had an atypical lying feature score of 7.85 (*S.E*. = 0.07, *p* < 0.001), falling near the mean and bottom of the subscale's range (5–20). This score significantly decreased into adulthood (*M* = −0.22, *S.E*. = 0.01, *p* < 0.001), though the decrease had significant deceleration (i.e., less decrease) over time and would level off (i.e., stop decreasing) around age 31 (*M* = 0.02, *S.E*. = 0.01, *p* < 0.001). Additionally, there was significant variance in the starting values, growth, and deceleration of individuals across time (all *p* < 0.001), suggesting significant sample heterogeneity in the lying trajectory.

**Figure A2:**
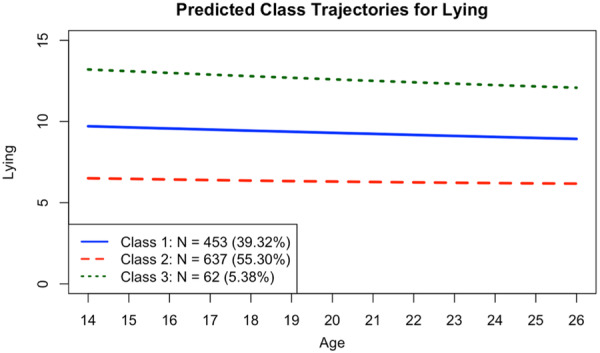
Predicted class trajectories for lying. *N* = 1152; RStudio (R Core Team, 2021); Class 1 = Moderate Lying Group Trajectory, Class 2 = Low Lying Group Trajectory, Class 3 = High‐Chronic Lying Group Trajectory.

Results of the six different latent class models, varying from one to six classes with quadratic growth specified for each, are depicted in Table A3. Results indicated that the model with three classes provided the best fit with high model classification certainty (entropy = 0.86; average posterior probabilities of belonging to each class >0.90). The model consisted of a low class who generally did not endorse our conceptualization of atypical lying (55.30%; intercept = 6.31), a moderate class with slightly above‐average atypical lying scores (39.32%; intercept = 9.31), and, supporting our hypothesis, a small high‐chronic class that consistently endorsed high levels of atypical lying features (5.38%; intercept = 12.62). The mean stability over time was high for each class, despite a slight decreasing trend for the higher classes, with final class trajectories depicted in Figure A2. Three individuals shifted from the moderate to the high class and one from the low class to the moderate class when completing the third LCGA step that included the other variables, but the growth factor (i.e., intercept, linear, quadratic) values remained unchanged.

### 3.2 Predictors and outcomes of lying trajectories

**Figure A3:**
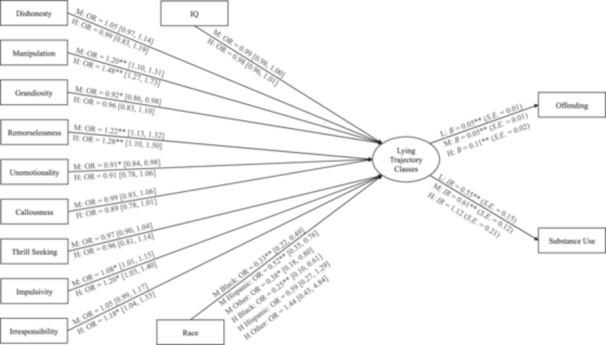
Structural Equation Model of Predictors and Outcomes of Lying Trajectories. *N* = 1152; Predictors of classes are compared to the low class; Outcome variables display unstandardized coefficients (if applicable) and standard errors. Not shown in this figure are the baseline controls for the offending and substance use outcomes. *p < 0.05, **p < 0.01. H, High‐Chronic group; IR, incident rate; L, Low group; M, Moderate group; OR, odds ratios with 95% confidence intervals in parentheses.

Comparisons of the third LCGA step, with predictors and outcomes, to its null version revealed that the correlated model had better relative fit (Loglikelihood = −53,842.35, AIC = 108,082.69, BIC = 109,087.50, SABIC = 108,455.41) than its uncorrelated counterpart (Loglikelihood = −54,127/75, AIC = 108,625.50, BIC = 109,559.61, SABIC = 108,971.99). Results of the final model are depicted in Figure A3 and as hypothesized, some interpersonal, affective and lifestyle traits were related to more risk. Specifically, compared to the low class, higher manipulation, remorselessness, and impulsivity scores in adolescence increased the odds of belonging to both elevated trajectories. Additionally, higher irresponsibility scores increased the odds of belonging to just the high‐chronic trajectory. Interestingly, higher values of grandiosity and unemotionality, and being nonwhite, significantly decreased the odds of belonging to the moderate trajectory group, while being Black decreased the odds of belonging to the high‐chronic group. The remaining predictors were not significant.

As expected, youth in the high‐chronic class engaged in significantly more types of offending in early adulthood compared to the other classes (both *p* < 0.002), while the low‐ and moderate‐classes did not significantly differ from each other. Furthermore, the low‐ and moderate‐classes significantly predicted less substance use at the final wave in early adulthood while the high‐chronic class used significantly more substances than both other classes based on parameter comparisons (both *p* < 0.003). Of the control variables, only baseline offending and substance use predicted their respective outcomes at the final wave (both *p* < 0.001).

## 4 DISCUSSION

Despite most people lying in some capacity (Vrij, 2000), normative developmental trends of lying frequency increase into adolescence, before decreasing into adulthood (Talwar & Crossman, 2011). However, around 5% of the population does not follow this normative desistance pattern and can become prolific liars (Curtis & Hart, 2022b). As hypothesized, we similarly observed an overall decrease in our conceptualization of atypical lying features (i.e., lying for fun or no reason, getting into trouble for excessive lying) into adulthood and that around 5% of the sample maintained elevated scores. This may indicate that the YPI lying subscale correlates, or indirectly measures, lie‐telling frequency because favorable attitudes towards lying are strong predictors of the behavior (Buta et al., 2021; Hart et al., 2019) and because the items capture those who are doubly rewarded for lying (i.e., gain from the lie plus enjoyment of it). However, the YPI in our LCGA could also be why we did not find a bigger chronic lying group despite examining a justice‐involved sample displaying elevated psychopathic traits. Specifically, although less practical than asking participants how many lies they tell, our robust longitudinal methodology may capture a more chronic, severe, and clinical level of atypical lying that identifies those most at risk. In this sense, our sample would hypothetically have more than 5% meet the prolific lying standard if it were based on frequency.

Regardless, those in the high trajectory group were more at risk for adulthood substance use and offending. Thus, by examining these atypical features of lying, we are able to identify at‐risk individuals and more specifically interpret etiological findings. Since higher manipulation and impulsivity scores were associated with elevated lying trajectories, atypical lying features seem to be both thought‐out and manipulative or a thoughtless inhibitory control issue. For the former, although manipulation is definitionally part of lying (Hart, 2019), it could be that some individuals enjoy being manipulative through lies. For the latter, impulsivity may limit the perceived punishment of a lie, ultimately turning lying into a “go‐to” strategy driven by the immediate rewards of dishonesty that mirror addictive neural responses. In this sense, prolific lying could be seen as an out‐of‐control compulsive craving for benefits. Alternatively, these results could indicate the presence of prolific and pathological liars in our sample, with a review showing that pathological liars do not lie maliciously, but instead often do so without incentive or reason (Curtis & Hart, 2022b). This may indicate that pathological liars are more impulsive than their manipulative counterparts who do not feel distress from their lies.

Next, the nonsignificant results for dishonest charm may imply that atypical lying features are not effective to con or seduce others over time. Alternatively, perhaps dishonest charm is related to more incentive‐based motives since the act requires effort that is not enjoyable on its own. Moreover, since irresponsibility (e.g., lack of punctuality) plays a larger role in atypical lying features than thrill seeking, some prolific liars may not lie for the excitement of avoiding detection, but rather because of their broader perceptions of social norms. Specifically, this may indicate a developmental impairment in understanding the social appropriateness of antisocial behaviors, where individuals inappropriately view lying, substance use, and offending as amusing rather than norm breaking.

Furthermore, the remorselessness results may signify that atypical lying features are due to not caring enough to tell the truth or the associated consequences of getting caught, which may increase the perceived enjoyment and utility of lying. Unexpectedly, higher unemotionality levels indicated lower odds of belonging to the moderate group compared to the low group, and callousness was not a significant predictor of membership to either elevated trajectory compared to the low group. Thus, those who do not get as anxious, nervous, or scared were less likely to endorse atypical lying features, and those who may not feel empathy towards others for lying did not display chronic elevations. These results could be due to our conceptualization of atypical lying or again differences between prolific and pathological liars. Specifically, unlike individuals high on psychopathic traits who lie, pathological liars experience guilt, remorse, and lack shallow affect (Curtis & Hart, 2020, 2022a). Nevertheless, higher grandiosity scores predicted lower odds of belonging to the moderate class compared to the low class. This suggests that those who do not view themselves as highly are more likely to endorse atypical lying features, potentially through the increased perceived rewards of having others seeing them through a more favorable, fantastical lens. This would resemble features of vulnerable narcissism (Miller et al., 2011), but more work is needed to understand this relationship. Regardless, grandiosity, unemotionality, and irresponsibility results should be interpreted with caution given their inconsistency in significance across both elevated classes.

For covariates, findings that White youths were more likely to belong to elevated lying trajectory groups could be a result of racial differences surrounding the justice system, social norms, and lying motives. For example, perhaps White youths believe they are more likely to get away with lying, which makes it more fun through a lack of fear for consequences. Next, although some argue that a higher IQ is needed to creatively manipulate and manage both the truth and lie at once (Talwar, 2018), having a lower IQ may be related to an inability to eloquently bend the truth or having more difficulties with concealing deceitful comments, leading to more explicit lying approaches. Our nonsignificant results may imply that both are true and cancelling each other out, or that IQ is not specifically related to our conceptualization of atypical lying features. Regardless, more work is needed to better understand the impact of these covariates.

This study is not without limitations and consequential future directions. In terms of the YPI, including all the subscales, some of which had poor reliability (e.g., callousness), to predict another subscale from the YPI (i.e., lying) may inflate or deflate the strength of the relationship between subscales (e.g., suppressor effect), despite a lack of multicollinearity issues (i.e., all tolerance values > 0.25; James et al., 2017). Additionally, the lying subscale does not cover all types of lying, fails to directly capture lying frequency, and is usually only administered to children at least 12 years old. Thus, our results may not generalize to all types of, or motives for, lying, and may not be applicable for younger ages when prolific lying first emerges. Moreover, the YPI cannot be experimentally manipulated, and thus causality was not established.

In terms of demographics, results are limited to male juvenile offenders who are likely more elevated on measures of psychopathic traits, lying, offending, and substance use than the general population. Additionally, we cannot generalize our results to ages not captured in our study and thus determine if atypical lying features follow a life‐course pattern. Moreover, as with all self‐reported measures of lying, we cannot be certain that those who report atypical lying features are not lying, especially about sensitive topics (i.e., substance use, offending). However, studies have found alignments between self‐report measures of deviant behavior (e.g., lying) and both polygraph and laboratory comparisons (Clark & Tifft, 1966; Green, 1990; Halevy et al., 2014). Lastly, our models did not control for important structural aspects related to our outcomes (e.g., socioeconomic status, institutionalized racism).

Despite these limitations, the present study is the first to use a longitudinal person‐centered approach to understand the development of atypical lying features across a critical normative desistance period that bridges childhood and adulthood behaviors. Using a large sample, we examined trends of lying across a developmental span of 13 years while accounting for both predictors and outcomes. Additionally, our measure of lying went beyond frequency in highlighting an atypical inclination for dishonesty through motives and attitudes that appear to match developmental patterns of lie‐telling trends. This indicates that targeting these more favorable lying beliefs before adolescence could be helpful in reducing lying's proclivity to become a “go‐to” strategy into adulthood.

Future studies should investigate the role of other individual (e.g., perception of social norms), cognitive (e.g., executive functions), environmental (e.g., parenting, peers), and biological factors (e.g., reactivity), as well as their interactions, to determine how lying becomes problematic. Specifically, researchers should work to further differentiate excessive liars' etiologies (i.e., prolific vs pathological). Lastly, more research is needed regarding the mechanisms that link individual traits to atypical lying features and the consequential outcomes. For example do atypical lying features lead to substance use through a loss of social credibility that jeopardizes relationships and limits social support, ultimately causing isolation and a negative self‐image? Does the consistent breaking of social norms by lying lead to an acceptance of, or desensitization to, antisocial behaviors?

## 5 CONCLUSION

The present study identified predictors of a high, sustained trajectory of atypical lying features, as well as ensuing risks (offending, substance use), during the normative developmental desistance period of lying (adolescence to adulthood). Prevention and intervention programs will want to target manipulative, remorseless, and irresponsible tendencies that may lead some to view lying as more favorable (i.e., fun, enjoyable), as well as impulsive traits that may impede cognitive rationalizations for honesty and lead to lying for no reason. Perhaps teaching perspective taking through ToM and problem‐solving skill will aid youth in understanding the harm that lying can cause and help them take the time to evaluate the appropriateness of different solutions to their problems. Specifically, given findings from Talwar et al. (2015) showing an ability to increase children's honesty with motivational rewards targeting the moral domain of Bandura's social cognitive theory (Bandura, 1986, 1991), findings from this study are particularly salient for identifying children who may benefit from programs that help develop self‐evaluative motivations through social incentives and the removal of competing social barriers for honesty. Lastly, children who display atypical lying features may benefit from substance use and crime prevention programs given their increased risk for these outcomes.

## CONFLICT OF INTEREST STATEMENT

The authors declare no conflict of interest.

## Data Availability

The data from the publicly available dataset data set used can be downloaded at https://www.icpsr.umich.edu/web/NAHDAP/studies/29961.

## 1

**Table A1:** Youth psychopathy inventory subscale descriptive and reliability.

	Mean	Standard deviation	Cronbach's alpha
Predictors (Mean of Waves 1 and 2)			Wave 1 | Wave 2
Dishonest charm (*N* = 1120)	10.31	3.35	0.81 | 0.81
Grandiosity (*N* = 1119)	10.92	2.80	0.66 | 0.63
Manipulation (*N* = 1119)	9.77	3.36	0.83 | 0.85
Remorselessness (*N* = 1120)	9.93	2.78	0.70 | 0.70
Unemotionality (*N* = 1120)	11.35	2.67	0.61 | 0.57
Callousness (*N* = 1120)	12.09	2.30	0.44 | 0.38
Thrill seeking (*N* = 1120)	13.34	2.85	0.66 | 0.63
Impulsivity (*N* = 1120)	11.48	2.99	0.64 | 0.70
Irresponsibility (*N* = 1120)	10.40	2.93	0.63 | 0.63
Lying subscale			
Wave 1 (*N* = 927)	9.04	3.37	0.79
Wave 2 (*N* = 1085)	8.57	3.28	0.81
Wave 3 (*N* = 1055)	8.42	3.26	0.83
Wave 4 (*N* = 1055)	8.70	3.30	0.82
Wave 5 (*N* = 1055)	8.21	3.04	0.80
Wave 6 (*N* = 1054)	8.21	3.05	0.81
Wave 7 (*N* = 1037)	7.84	2.93	0.81
Wave 8 (*N* = 1025)	7.60	2.85	0.81
Wave 9 (*N* = 1001)	7.34	2.66	0.81
Wave 10 (*N* = 958)	7.49	2.68	0.82

*Note*: Range of all subscales = 5–20.

**Table A2:** YPI subscale, inter‐subscale correlation matrix with Holm‐Bonferroni adjustment.

		1.	2.	3.	4.	5.	6.	7.	8.	*9*.	*10*.
1.	Dishonest charm	‐									
2.	Grandiosity	0.55**	‐								
3.	Lying	0.56**	0.30**	‐							
4.	Manipulation	0.83**	0.54**	0.63**	‐						
5.	Remorselessness	0.59**	0.43**	0.53**	0.63**	‐					
6.	Unemotionality	0.51**	0.51**	0.38**	0.52**	0.61**	‐				
7.	Callousness	0.06*	−0.00	−0.02	0.14**	0.18**	0.17**	‐			
8.	Thrill seeking	0.53**	0.40**	0.46**	0.55**	0.57**	0.51**	0.02	‐		
9.	Impulsivity	0.53**	0.35**	0.53**	0.54**	0.59**	0.47**	0.03	0.67**	‐	
10.	Irresponsibility	0.46**	0.31**	0.46**	0.46**	0.50**	0.39**	0.10**	0.48**	0.54**	‐

*Note*: Variables are mean of waves 1 and 2; Manipulation and grandiosity: *N* = 1119; Other means: *N* = 1120.

* *p* < 0.05;

** *p* < 0.01.

**Table A3:** Results from Step 1 of the lying quadratic latent class growth analyses (ages 14–26).

	Log‐likelihood	AIC	BIC	SABIC	Entropy	Largest class	Smallest class
1 Class	−25,910.68	51,847.36	51,913.00	51,871.71	1.00	100%	‐
2 Classes	−24,544.07	49,122.13	49,207.97	49,153.97	0.856	62.85%	37.15%
**3 Classes**	**−24,245.38**	**48,532.76**	**48,638.79**	**48,572.09**	**0.868**	**55.38%**	**5.12%**
4 Classes	−24,117.30	48,284.60	48,410.83	48,331.42	0.803	42.71%	2.17%
5 Classes	−24,038.28	48,134.57	48,280.99	48,188.88	0.777	43.66%	2.26%
6 Classes	−23,990.30	48,046.61	48,213.23	48,108.41	0.772	42.62%	1.91%

*Note*: *N* = 1152; Largest and smallest class column represent percentage of the sample included Bolded line was the best fitting model.
